# Profiling of the gut, skin and nasal microbiotas revealed clinically relevant microbial taxa from children with allergies: a pilot study

**DOI:** 10.3389/falgy.2025.1497914

**Published:** 2025-02-27

**Authors:** Jiayi Hong, Zhiwei Tang, Dongjun Zhang, Chenqi Mo, Wen Su, Jie Shao

**Affiliations:** Department of Paediatrics, Ruijin Hospital Affiliated with Shanghai Jiaotong University School of Medicine, Shanghai, China

**Keywords:** food allergy, atopic dermatitis, allergic rhinitis, gut microbiota, nasal microbiota, skin microbiota

## Abstract

**Background:**

A reduction in biodiversity and alterations in the microbiota composition are relevant to allergic diseases. However, combined analyses of the skin, nasal and gut microbiotas are lacking in the literature. In addition, in previous studies, microbiota were detected mainly by V3–V4 sequencing, but other sequences might be missed with this technique.

**Methods:**

In this case–control study, we enrolled 3–12-year-old children with allergic rhinitis combined with atopic dermatitis and food allergy (AR-AD-FA group), children with allergic rhinitis only (AR-only) and healthy controls (HC group). We employed full-length 16S rRNA gene amplification and sequencing for the detection of gut, nasal and skin microbiota.

**Results:**

Samples with an average sequence length of 1,459 bp were obtained in this study. Significant differences in beta diversity in the three compartments were found between the disease groups and the HC group. Differentially expressed genera were present mainly in the gut compartment. *Peptoniphilus, Prevotella* and *Anaerococcus* were abundant in the gut in the disease groups. Specifically, *Streptomyces, Thermus* and *Pseudomonas* showed differential expression in both the nasal and skin compartments of children in the disease groups.

**Conclusion:**

Some meaningful differences in the abundance of some microbiota from the three compartments were observed between the disease groups and the HC group. These findings could provide new insights into the prevention and treatment of allergic diseases through the regulation of specific microbiota in the future.

## 1 Introduction

The prevalence of allergic diseases has continued to increase across developing countries in recent years (1). The biodiversity hypothesis suggests that a reduction in biodiversity and alterations in microbiota composition are relevant to allergic diseases and that exposure to nonpathogenic commensal bacteria could regulate immunity to some extent (1). Therefore, identifying the specific microbiota features associated with allergic diseases might provide novel mechanisms and targets, thus improving the prevention and treatment of allergic diseases.

The gut, skin, and airway harbour the most abundant microbiota in the human body (2, 3). Recent studies have shown that the microbiota located in the gut, skin and airway play important roles in both human health and disease. The composition and structure of the gut flora reach a stable state at 2–3 years of age (4), and the skin flora is similar to that of adults at 12–18 months of age (5). Major changes in the airway flora always occur in the year following birth (6).

The gut‒skin axis theory suggests that disruptions in gut barrier integrity and imbalances in the gut microbial community could significantly impact skin homeostasis (7). Changes in the composition of the gut microbiota due to diet, disease, and medications are associated with alterations in airway homeostasis, which supports the concept of the “gut‒lung axis” (8). However, most of the previously published research has studied the gut, skin, and airway microbiota separately instead of studying all three compartments as an ecological community.

The progression from atopic dermatitis (AD) and food allergy in infancy to asthma and allergic rhinitis (AR) in childhood is known as the “allergic march”. Several studies have found that alterations in the microbiota can influence the allergic process as well. It has been proposed that early skin dysbiosis is more likely to lead to the development of AD, which can be corrected and improved with colonization by commensal *staphylococci* (9). Furthermore, patients with decreased gut flora diversity in infancy have been shown to be at increased risk of developing atopic diseases (10–12). To our knowledge, few studies have compared microbiota differences in children with AR with or without food allergy (FA) and AD.

In recent years, 16S rRNA gene sequencing has been widely used for the identification of microbiota because of its widespread distribution, high abundance and low detection cost. Currently, the V1–V3 variable region sequences of 16S rRNA are used to identify skin microbiota (13, 14), and the V3–V4 variable region sequences are used to identify gut microbiota (13, 15). In previous studies evaluating both skin and gut compartments, microbiota were detected via V3–V4 sequencing (13). Therefore, it is possible that specific skin microbiota expressing other sequences might be missed. To avoid missing important microbiota, full-length 16S rRNA gene sequencing of microbiota was employed in our study.

In this study, we explored the differential expression of microbiota in children with different allergies and delineated the clinical relevance of the gut, skin, and nasal microbiota to the allergy phenotype in children with allergic rhinitis combined with atopic dermatitis and food allergy (AR-AD-FA group), children with allergic rhinitis only (AR-only group) and healthy controls (HC group).

## 2 Methods

### 2.1 Subjects

Children aged 3–12 years treated for FA, AD or AR from December 2019 to January 2021 at the paediatric outpatient clinic of Ruijin Hospital were included in the disease groups. Fifteen age- and sex-matched healthy children were recruited as controls. The inclusion criteria for the subjects were as follows: (1) aged between 3 and 12 years; (2) diagnosed with AD and FA; and (3) diagnosed with AR. The exclusion criteria were as follows: (1) had asthma; (2) had diarrhoea or constipation in the previous months; (3) had used antibiotics or probiotics in the previous months; (4) had received immunotherapy; (5) had respiratory infections, gastrointestinal infections or skin infections; and (6) had other immune disorders or gastrointestinal disorders. HC had no history of atopic disease. The exclusion criteria for the controls were the same as those for the subjects. Ultimately, 9 patients with AR combined with AD and FA, 12 patients with AR only, and 15 HCs were included in the study. The study was approved by the Ethics Committee of Ruijin Hospital, and informed consent forms were signed by the guardians of all participants.

### 2.2 Questionnaires

This study employed the same questionnaire that was applied in the China, Children, Homes, Health (CCHH) programme (16). The questionnaire included questions on individual characteristics, such as sex, age, birth history, feeding history, term birth status, birth weight, mode of delivery, and family characteristics, such as parental education level and history of allergy. Questions on factors that might increase the risk of FA and AR were also considered in the questionnaire.

### 2.3 Diagnosis of AD

AD was diagnosed according to the Williams criteria (17). Patients who met at least 3 of the major criteria were diagnosed with AD.

### 2.4 Diagnosis of FA

The diagnosis of FA was made according to the criteria outlined in the European Academy of Allergy & Clinical Immunology (EAACI) Food Allergy Guidelines (18).

### 2.5 Diagnosis of AR

The diagnosis of AR was made according to the criteria outlined in the Clinical Practice Guidelines for Diagnosis and Treatment of Allergic Rhinitis in Pediatrics (19).

All children were diagnosed by paediatric allergists who had more than 5 years of experience in the diagnosis and treatment of allergic diseases.

### 2.6 Sample collection

Fecal samples were collected from selected children. Skin flora samples were collected by rubbing nonlesional skin behind the ear with a sterile swab for 10 s. Nasal flora samples were collected by rotating a swab in the nose 10 times. All the collected samples were stored at −80°C.

### 2.7 Experimental procedure

#### 2.7.1 Sample DNA extraction

Sample microbial DNA was extracted with an OMEGA Soil DNA Kit (M5635-02) (Omega Bio-Tek, Norcross, GA, USA), and the extracted DNA was stored at −20°C. The quantity and quality of the extracted DNA were assessed with a NanoDrop NC2000 spectrophotometer (Thermo Fisher Scientific, Waltham, MA, USA) and electrophoresis on a 1% agarose gel.

#### 2.7.2 16s rRNA gene amplification and sequencing

The forward primer 27F (5′-AGAGTTTGA TCMTGGCTCAG-3′) and reverse primer 1492R (5′-ACCTTGTTACGACTT-3′) were used for PCR amplification of the full-length bacterial 16S rRNA gene. The extracted DNA was amplified via two-step PCR, and the sample-specific 16 bp barcode was integrated into the forwards and reverse primers for multiple sequencing in the second PCR step. All the PCR amplicons were purified with Agencourt AMPure Beads (Beckman Coulter, Indianapolis, IN) and quantified with a PicoGreen dsDNA Assay Kit (Invitrogen, Carlsbad, CA, USA). After the individual quantification step, equal amounts of amplicons were pooled. Single-molecule real-time (SMRT) sequencing was performed with the PacBio Sequel platform from Shanghai Parsonage Biotech (Shanghai, China).

#### 2.7.3 Analysis of sequencing data

Sequencing data analyses were performed via a platform named “personalbio”, which provides statistic analysis via QIIME2 and the R software (v4.1.1). The relative abundance at the genus level is presented in stacked histograms. Generally, alpha diversity reflects the variety of microbial species within a single sample, indicating how diverse the microbial community is in that particular environment. In contrast, beta diversity compares the microbial composition between different samples, highlighting how the microbial communities differ from one sample to another. Alpha diversity indices, including the Chao1, observed species, and Shannon and Simpson indices, were calculated with QIIME2 software and visualized in box plots. The beta diversity comparison was evaluated via principal coordinate analysis (PCoA) on the basis of the Bray‒Curtis distances. Differential microbiota between groups were identified via linear discriminant analysis (LDA) and LDA effect size (LEfSe) with an LDA threshold of 2 to identify biomarkers (20).

### 2.8 Statistical analyses

Clinical data were analysed via SPSS 26.0, with an alpha level of 0.05. *P* values were false discovery rate (FDR)-corrected for multiple comparisons. The quantitative data were analyzed via *t* tests or analysis of variance (ANOVA), whereas the qualitative data were analyzed via the chi-square test or Fisher's exact test.

## 3 Results

### 3.1 Baseline characteristics of the participants

Ultimately, 9 patients with AR combined with AD and FA (AR-AD-FA group), 12 patients with AR only (AR-only group) and 15 HCs (HC group) were included in this study. There were no statistically significant differences in the participants' individual or family baseline characteristics (Table 1).

**Table 1:** Comparison of the demographic information of the enrolled groups.

Parameter	AR-AD-FA	AR-only	HC	*P*
	*N* = 9	*N* = 12	*N* = 15	>0.05
	(7/9)	(9/12)	(15/15)	
Gender				
Male	5/7 (71.4%)	5/9 (55.6%)	9/15 (60%)	>0.05
Female	2/7 (28.6%)	4/9 (44.4%)	6/15 (40%)	
Age at enrollment (months), mean ± SD	75 ± 37.3	89 ± 22.4	68.7 ± 28.2	>0.05
Weight at birth(g), mean ± SD	3,464 ± 289.7	3,511 ± 553.8	3,400 ± 388.7	>0.05
Full term delivery	6/7 (85.7%)	8/9 (88.9%)	11/15 (73.3%)	>0.05
C-section	3/7 (42.9%)	5/9 (55.6%)	5/15 (33.3%)	>0.05
Duration of breast-feeding (months), mean ± SD	4.42 ± 1.8	4.78 ± 1.7	4.0 ± 1.8	>0.05
Place of living				
Urban	4/7 (57.1%)	7/9 (77.8%)	8/15 (53.3%)	>0.05
Suburbs	3/7 (42.9%)	2/9 (22.2%)	7/15 (46.7%)	>0.05
Parents' education				
Mother Secondary	7/7 (100%)	7/9 (77.8%)	9/15 (60.0%)	>0.05
Mother Higher	0 (0%)	2/9 (22.2%)	6/15 (40.0%)	
Father Secondary	6/7 (85.7%)	8/9 (88.9%)	7/15 (46.7%)	>0.05
Father Higher	1/7 (14.3%)	1/9 (11.1%)	8/15 (53.3%)	
Atopy in family				
Father	1/7 (32.3%)	1/9 (11.1%)	3/15 (20.0%)	>0.05
Mother	1/7 (32.3%)	1/9(11.1%)	5/15(33.3%)	>0.05

### 3.2 Sample analysis results

We obtained the nucleotide sequences of 102 samples, with an average sequence length of 1,459 bp.

### 3.3 Comparative results of the microbiota in different compartments

#### 3.3.1 Gut microbiota

**Figure 1:**
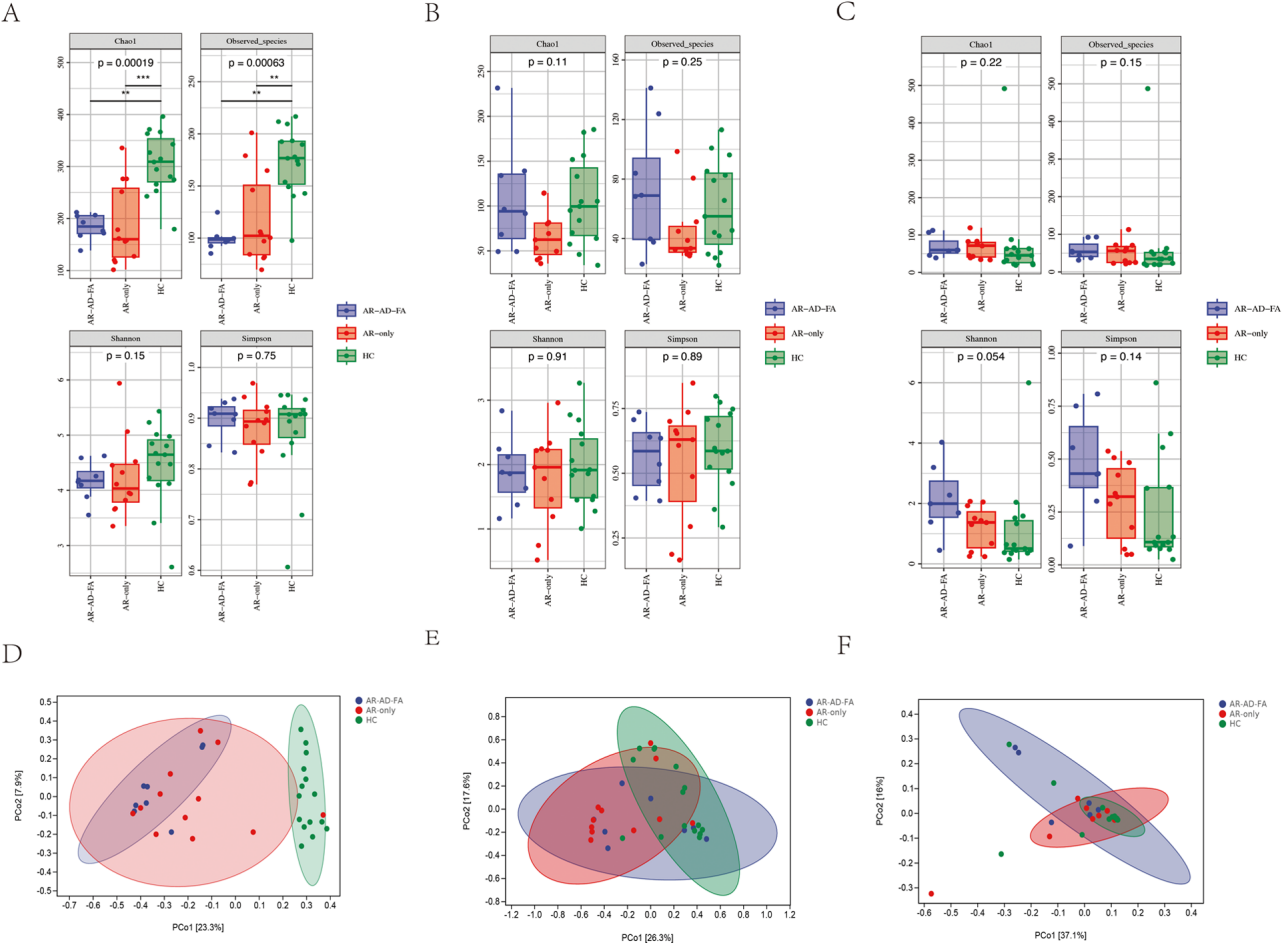
Alpha diversity of the gut **(A)**, nasal **(B)** and skin **(C)** microbiota in children in the AR-AD-FA, AR only and HC groups. The *p*-values annotated in the figure represent the *p*-values of the three group comparisons. Beta diversity of the gut **(D)**, nasal **(E)** and skin **(F)** microbiota in children in the AR-AD-FA, AR only and HC groups. F—faeces from the gut, N-nasal, E—the skin behind the ear, AR-AD-FA—allergic rhinitis combined with atopic dermatitis and food allergy, AR only—allergic rhinitis, HC—healthy controls.

The Chao1 index and observed species index of the gut microbiota of children in the AR-AD-FA and AR-only groups were significantly lower than those in the HC group. These findings suggested that the alpha diversity decreased in the disease groups, including the AR-AD-FA and AR-only groups (Figure 1A).

There were significant differences in the community structure of the gut microbiota among the AR-AD-FA, AR-only and HC groups (*p* = 0.001). In addition, differences in the community structure of the gut microbiota were observed between the AR-AD-FA and HC groups, as well as between the AR-only and HC groups (*p* = 0.015, corrected). Significant differences in beta diversity were observed between the disease groups and the HC group (Figure 1D).

At the genus level, the gut community was more abundant in *Prevotella, Peptoniphilus* and *Anaerococcus* in the disease groups than in the HC group. However, *Faecalibacterium, Bacteroides, Roseburia, Blautia* and *Mediterraneibacter* were more abundant in the HC group.

**Figure 2:**
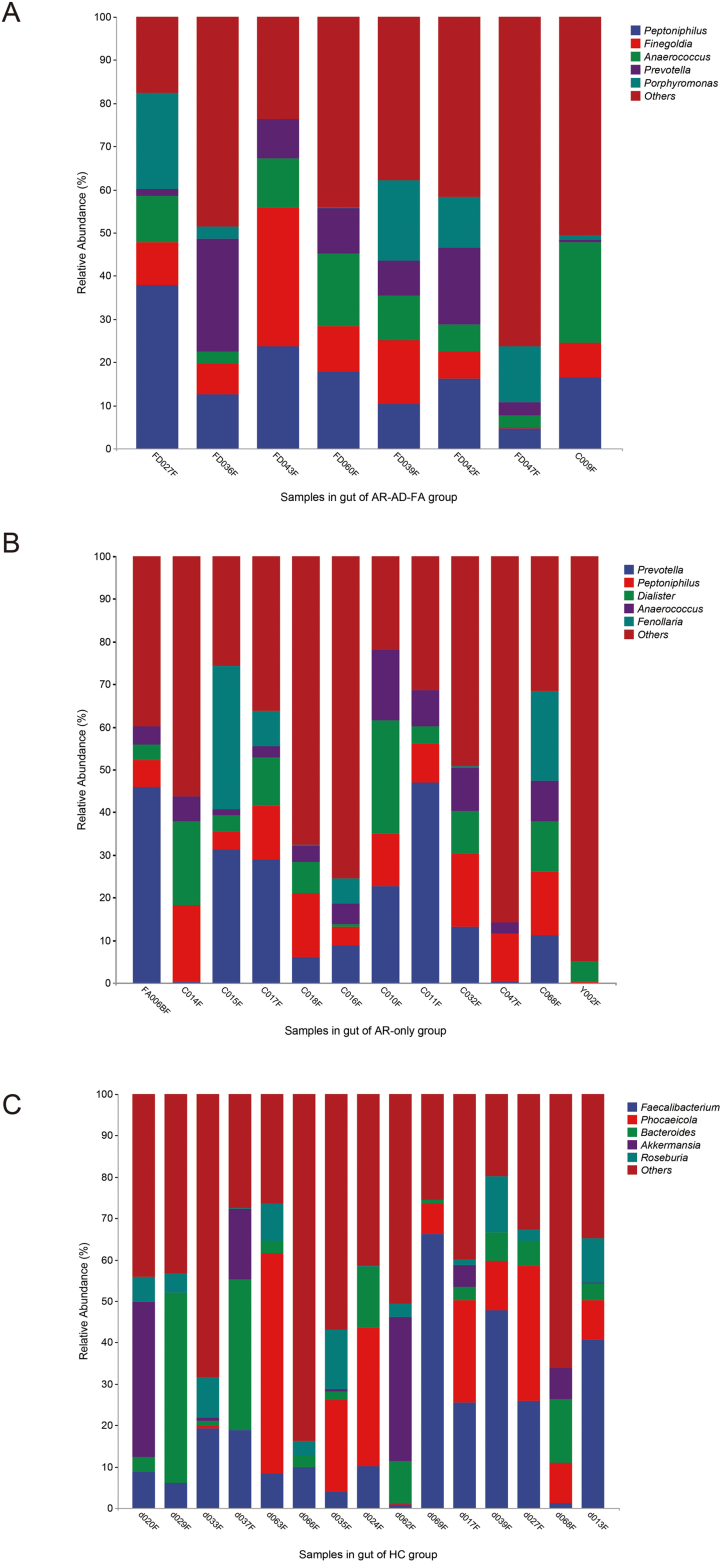
**(A–C)** The top five most abundant genera in the gut of the three groups (AR-AD-FA, AR only and HC groups). AR-AD-FA—allergic rhinitis combined with atopic dermatitis and food allergy, AR only—allergic rhinitis, HC—healthy controls.

*Peptoniphilus, Anaerococcus* and *Prevotella* were abundant in the gut microbiota of both the AR-AD-FA group and the AR-only group (Figures 2A,B). In addition, *Finegoldia* and *Porphyromonas* were specifically abundant in the gut microbiota of the AR-AD-FA group (Figure 2A), whereas *Dialister* and *Fenollaria* were more abundant in the AR-only group (Figure 2B). *Faecalibacterium, Phocaeicola, Bacteroides, Akkermansia* and *Roseburia* were the top five most abundant genera in the HC group (Figure 2C).

**Figure 3:**
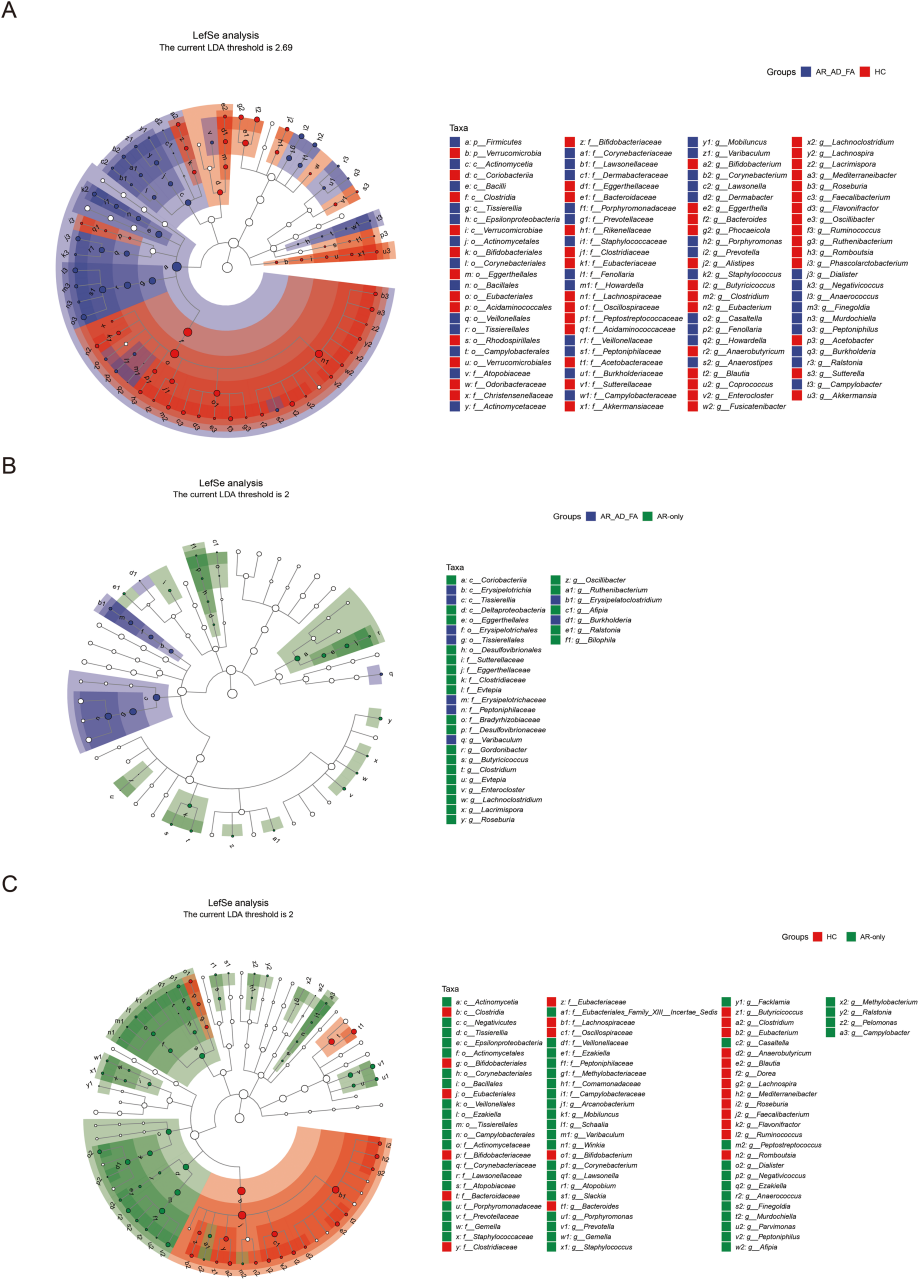
**(A–C)** The differentially expressed genera and pairwise comparisons among the three groups (AR-AD-FA, AR only and HC groups) from LefSe analysis of the gut are presented. F—faeces from the gut; AR-AD-FA—allergic rhinitis combined with atopic dermatitis and food allergy, AR only—allergic rhinitis, HC—healthy controls.

In the LEfSe analysis (LDA threshold of 2), the differentially expressed genera of the gut flora of the AR-AD-FA and AR-only groups were compared with those of the HC group (Figures 3A,B). After the genera that differed consistently between the two disease groups were removed (Figure 3C), *Dermabacter, Fenollaria, Howardella* and *Anaerostipes* were highly expressed specifically in the AR-AD-FA group, whereas *Burkholderia, Arcanobacterium, Schaalia, Winkia, Atopobium, Slackia, Gemella, Facklamia, Peptostreptococcus, Ezakiella, Parvimonas, Afipia, Methylobacterium* and *Pelomonas* were highly expressed specifically in the AR-only group. Furthermore, in the LEfSe analysis of th AR-AD-FA and AR-only groups, the differentially expressed genera were *Varibaculum, Erysipelatoclostridium, Burkholderia, Gordonibacter, Butyricicoccus, Clostridium, Evtepia, Lachnoclostridium, Roseburia, Oscillibacter, Ruthenibacterium, Afipia, Ralstonia, Enterocloster, Lacrimispora* and *Bilophila*.

#### 3.3.2 Nasal microbiota

There were no significant differences in alpha diversity between the disease groups and the HC group (Figure 1B). A significant difference in the beta diversity of nasal microbiota was observed between the AR-only group and the HC group (*p* = 0.006, corrected), which suggested there was a significant difference in the community structure of the AR-only group and the HC group, whereas no significant differences were detected between the two disease groups (Figure 1E).

**Figure 4:**
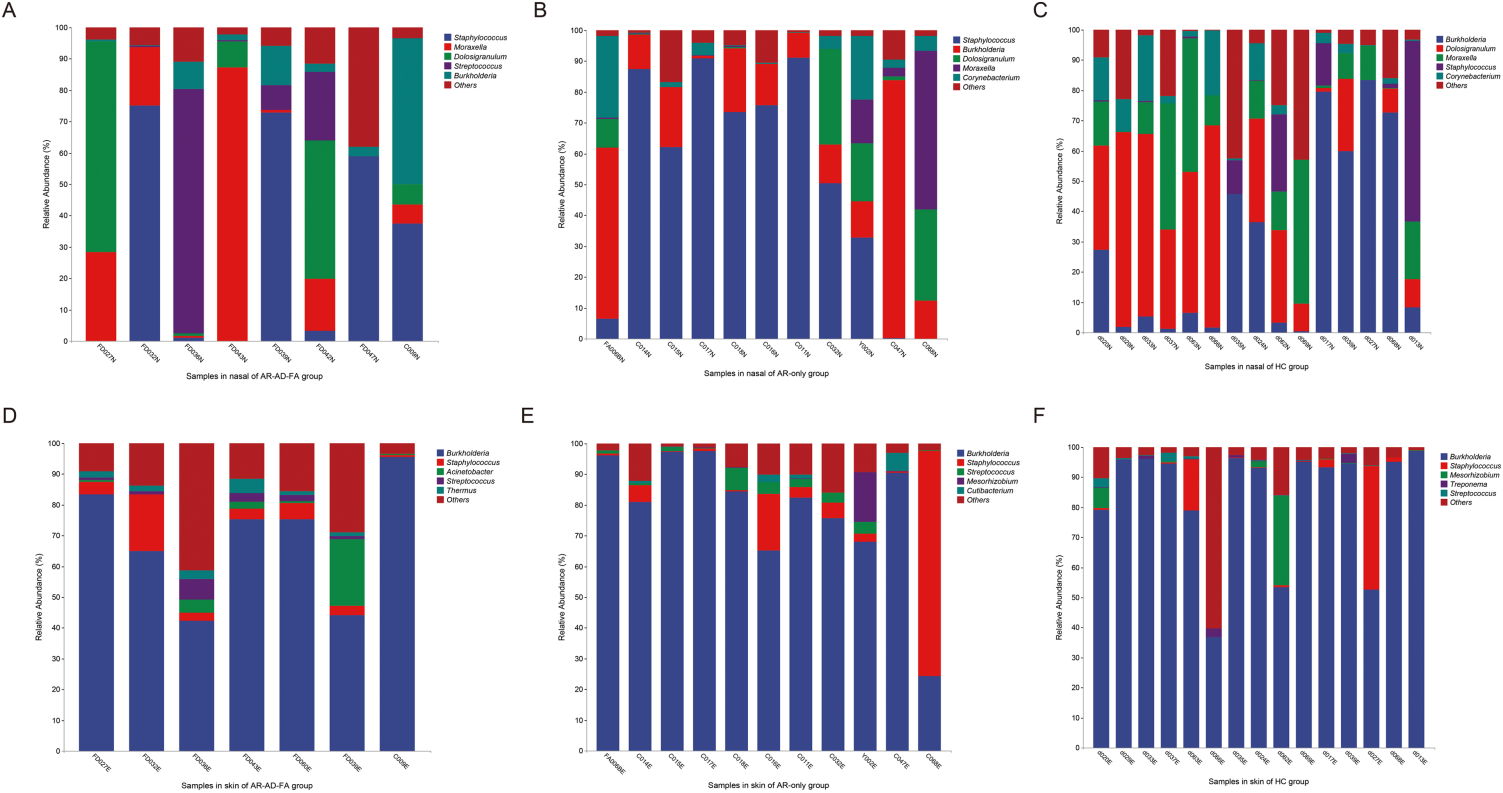
**(A–F)** The top five most abundant genera in two compartments (nasal and skin) of the three groups (AR-AD-FA, AR only and HC groups). AR-AD-FA—allergic rhinitis combined with atopic dermatitis and food allergy, AR only—allergic rhinitis, HC—healthy controls.

*Staphylococcus, Moraxella, Dolosigranulum* and *Burkholderia* were the top five most abundant genera in the nasal cavity and were observed in all the groups (Figures 4A–C). *Streptococcus* was specifically abundant in the nasal cavity of the AR-AD-FA group (Figure 4A).

**Figure 5:**
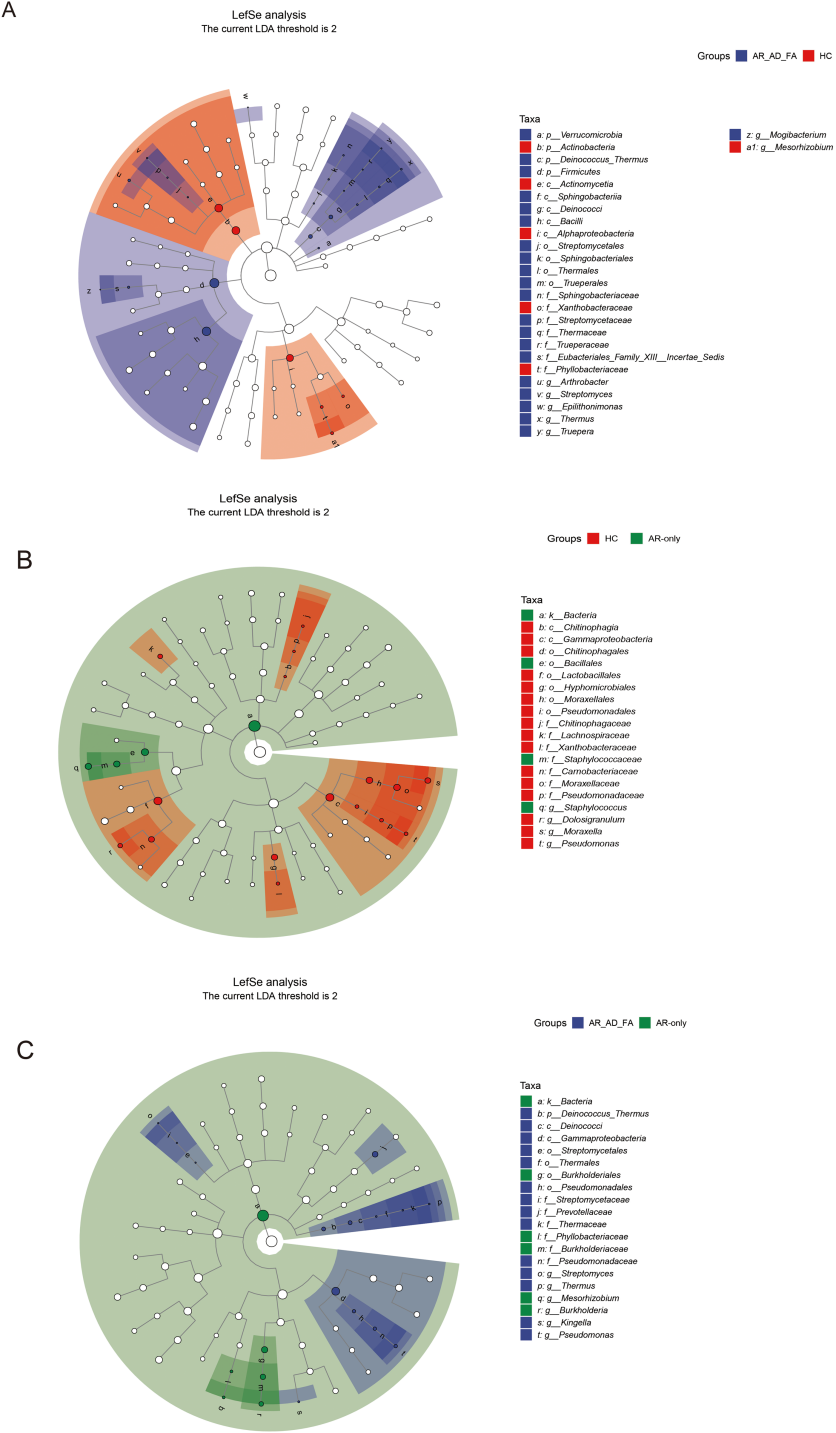
**(A–C)** The differentially expressed genera and pairwise comparisons among the three groups (AR-AD-FA, AR only and HC groups) from LefSe analysis of the nasal are presented. N-nasal; AR-AD-FA—allergic rhinitis combined with atopic dermatitis and food allergy, AR only—allergic rhinitis, HC—healthy controls.

The differentially expressed genera identified via LEfSe analysis of the nasal flora of the AR-AD-FA and AR-only groups were compared with those identified in the HC group (Figures 5A,B). After the genera that differed consistently between the two disease groups were removed (Figure 5C), *Streptomyces, Thermus, Kingella, Pseudomonas* and *Staphylococcus* were highly expressed specifically in the AR-AD-FA group. LEfSe analysis confirmed that *Streptomyces, Thermus, Kingella, Pseudomonas, Burkholderia* and *Mesorhizobium* were the differentially expressed genera between the two disease groups.

#### 3.3.3 Skin microbiota

There was no significant difference in alpha diversity between the disease groups and the HC groups (Figure 1C). However, a significant difference in the community structure of the skin samples was observed among the three groups (*p* = 0.046). Significant differences in beta diversity were observed among the three groups (Figure 1F). Furthermore, the comparisons between the two disease groups and the HC group revealed differences only between the AR-AD-FA and HC groups (*p* = 0.026).

*Burkholderia, Staphylococcus* and *Streptococcus* were the top five most abundant genera in the skin samples among the three groups (Figures 4D–F). *Treponema* was more abundant at the genus level within the skin community of the HC group. *Thermus, Pseudomonas* and *Halomonas* were more abundant in the AR-AD-FA group. There were significant differences in *Mesorhizobium* and *Rhodopseudomonas* abundance between the two disease groups.

**Figure 6:**
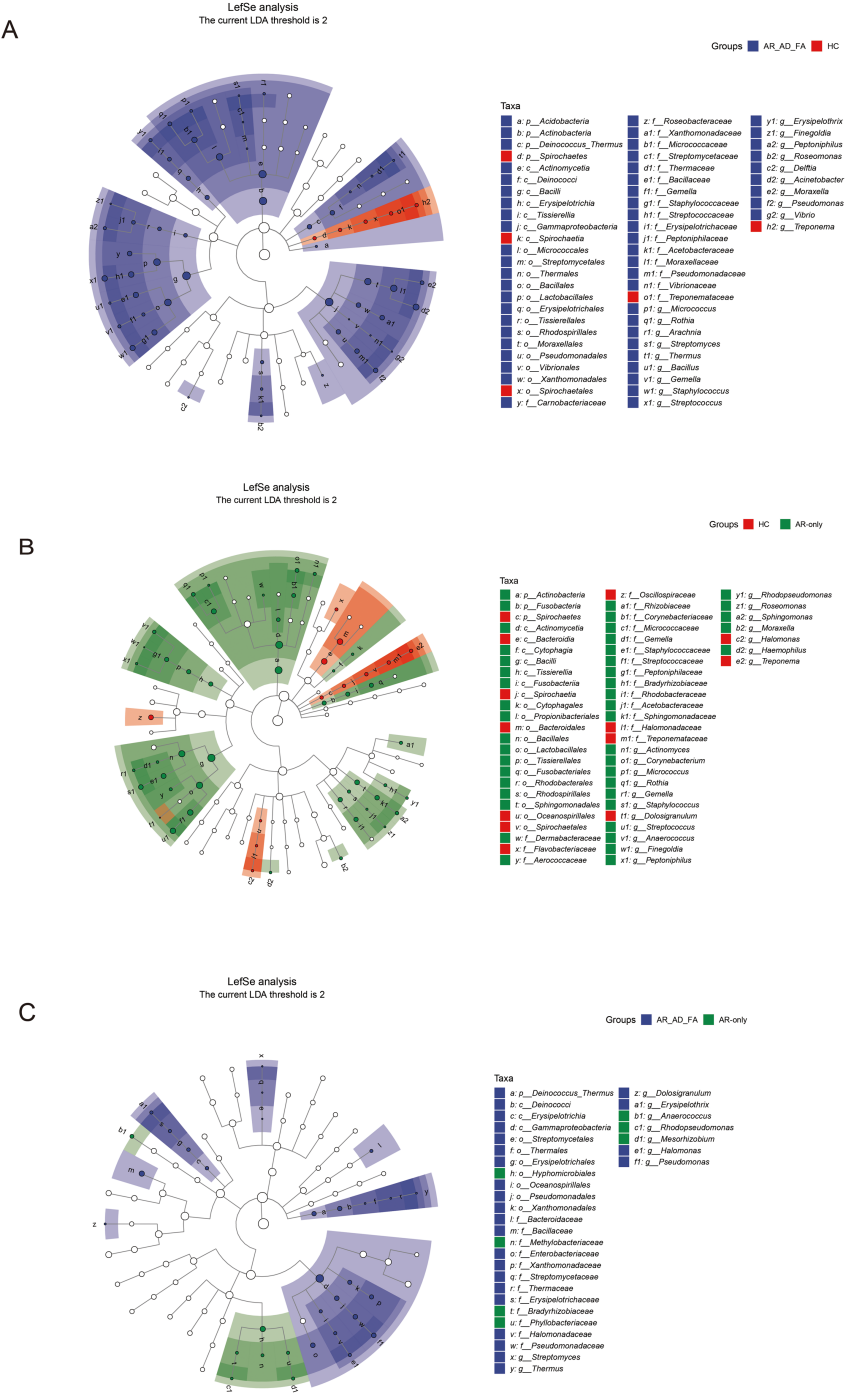
**(A–C)** The differentially expressed genera and pairwise comparisons among the three groups (AR-AD-FA, AR only and HC groups) from LefSe analysis of the skin are presented. E—the skin behind the ear; AR-AD-FA—allergic rhinitis combined with atopic dermatitis and food allergy, AR only—allergic rhinitis, HC—healthy controls.

In the LEfSe analysis, the differentially expressed genera of the skin flora of the AR-AD-FA and AR-only groups were compared with those of the HC group (Figures 6A,B). After the genera that differed consistently between the two disease groups were removed (Figure 6C), *Arachnia, Streptomyces, Thermus, Bacillus, Erysipelothrix, Delftia, Acinetobacter, Pseudomonas, Vibrio, Actinomyces, Corynebacterium, Anaerococcus, Rhodopseudomonas, Sphingomonas* and *Haemophilus* were highly expressed specifically in the AR-AD-FA group. *Streptomyces, Thermus, Dolosigranulum, Erysipelothrix, Halomonas, Pseudomonas, Anaerococcus, Rhodopseudomonas* and *Mesorhizobium* were considered differentially expressed genera between the two disease groups according to the LEfSe analysis.

## 4 Discussion

In our case‒control study, we compared the expression of genes associated with the gut, nasal and skin microbiota in children with AR combined with AD and FA, AR only and healthy children. Significant differences in beta diversity were found between the disease groups and the HC group in the three compartments. Differentially expressed genera were present mainly in the gut compartment. *Peptoniphilus, Prevotella* and *Anaerococcus* were abundant in the gut in the disease groups. *Streptomyces, Thermus,* and *Pseudomonas* were highly expressed in the nasal and skin compartments of children in the disease groups. To our knowledge, this study is the first to evaluate the gut, nasal and skin compartments to investigate the interactions among the microbiota in these compartments in individuals with allergic diseases. Furthermore, full-length 16S rRNA gene sequencing of microbiota was employed to avoid missing other sequences.

The alpha diversity of the gut microbiota was significantly lower in both disease groups than in the HC group, which was also consistent with previous findings (13). Previous studies have shown that gut microbiota maintain the balance between type 1 (Th1) and type 2 (Th2) T-helper cell immune responses by regulating various lymphocyte subpopulations, especially regulatory T (Treg) cells (4, 21–23). In the absence of gut microbiota, the levels of both IgE and circulating basophils tend to increase (24), thus leading to a Th1/Th2 cell immune response imbalance that could cause allergic symptoms.

However, the skin microbiota of the three groups did not differ significantly in terms of microbial alpha diversity, which was different from previous studies showing that patients with AD had reduced skin microbial diversity (25, 26). Possible reasons for this discrepancy between our study findings and those of previous studies were the differences in disease phenotypes, sampling sites and detection methods. Children with FA were enrolled in this study, whose AD was associated with FA. In other words, the severity of AD was significantly reduced after avoiding the allergic food. The relatively reduced Th1 cell immune response might result in the skin being chronically infected with bacteria. A previous study showed that patients with AD have greater biodiversity in nonlesioned areas than in lesioned areas, and the microbial diversity in lesioned areas gradually increases with therapeutic intervention (27). In the present study, the microbiota of nonlesional postauricular skin from patients with AD was sampled, amplified and sequenced with the full-length 16S rRNA gene, unlike in previous analyses of skin flora fragments.

Notably, we found that *Streptomyces, Thermus* and *Pseudomonas* were highly expressed in the nasal cavity and skin of patients with AR combined with AD and FA. To date, few studies on *Streptomyces* in patients with allergies have been reported. Lu et al. reported a significant increase in *Streptomyces* abundance in the skin lesions of patients with active vitiligo (28). *Streptomyces* can produce numerous secondary metabolites similar to the calcineurin inhibitor tacrolimus, which is widely used for the treatment of AD (29). The significantly increased abundance of *Streptomyces* in the skin and nasal cavities of patients with AR combined with AD and FA might constitute a self-regulatory mechanism of the skin or mucosa. However, the confirmed correlation between *Thermus* abundance and FA and AD remains unclear. A study focusing on the characterization of the ocular and nasopharyngeal microbiomes in individuals with allergic rhinoconjunctivitis revealed that *Thermus* was more abundant on the ocular surface than on the nasal surface, especially in healthy children (30). This difference might be attributed to the different phenotypes of the allergic diseases. *Pseudomonas* is an accepted pathogenic bacterium associated with allergic diseases. A high abundance of *Pseudomonas* during pregnancy is a risk factor for the development of AD (31). A higher abundance of *Pseudomonas* had been found in the gut of children with FA than in that of healthy children (32). Furthermore, a study showed that infants who had developed allergies at the age of 7 years had a significant increase of *Pseudomonas* abundance in breastmilk during the first month of life (33). *Peptoniphilus* abundance is high in the sinonasal cavity, and *Peptoniphilus* is always found in patients with chronic rhinosinusitis (34, 35); however, *Peptoniphilus* is seldom found in the gut. In contrast to our study, previous studies have observed *Prevotella* in rural healthy children and in children with a lower occurrence of FA (36, 37). Dietary prebiotics were found to promote intestinal *Prevotella* in a murine oxazolone-induced model of AD (38). Furthermore, fructo-oligofructose treatment was found to increase the relative abundance of *Prevotella* in the gut microbiota of mice with AD (39). In this study, participants with AD were in the stable phase, and the recovery process may have led to the increase in *Prevotella* abundance.

The direct associations between specific microbiota, including *Burkholderia, Afipia, Kingella* or *Rhodopseudomonas*, and allergic diseases, including FA, AD or the allergic march, have not been reported previously. *Burkholderia* belongs to the phylum *Proteobacteria*, with an outer membrane composed of lipopolysaccharides (LPS) (40). Elevated LPS could increase the risk of inflammation and immune abnormalities (41–43). The home environmental microbiota in children with eczema is characterized by a greater abundance of *Anaerococcus* than that in healthy children (44). In our study, *Anaerococcus* was significantly more abundant in the skin compartment of the AR-only group than in those of the AR-AD-FA and HC groups.

In the classic allergic march, allergic diseases progress from AD and FA in infancy to allergic respiratory diseases in childhood, such as AR or asthma, while the mechanisms underlying the allergic march remain to be fully investigated. Recent studies have revealed the role of the microbiome in the allergic march. On the basis of the current literature, our study is the first in which a systematic comparative analysis of the combined gut, nasal and skin microbiomes in allergic children in mainland China was performed.

The *V3*–*V4* hypervariable region of the 16S rRNA gene was analysed via high-throughput DNA sequencing in previous studies, reducing the detection cost, but some important microbiota in other sequences could be missed. To overcome this problem, we chose to amplify and sequence the full-length 16S rRNA gene of microbiota. The final average length of the analysed sequences was 1,459 bp, which was essentially comparable to the theoretical length of the full-length sequences and met the requirements for amplification and sequencing. We considered the characteristics of microbiota expressed in different compartments that were close to the accurate results and real conditions, which is another strength of this study.

However, there are also several limitations of this study. Our sample size was small, and the sample size should be increased in future studies to verify these findings. Future studies should consider disease state and severity when grouping patients. The lack of gut, nasal, and skin samples from early life limited our ability to verify the changes in the microbiota caused by allergic diseases. Further studies could be designed as cohort studies, and more than 2 years of follow-up are necessary.

## 5 Conclusion

The skin, nasal, and gut flora should be analysed in combination to investigate the differences in microbiota between children with and without allergies. Some important microbiota could be missed when sequencing only partial regions of the 16S rRNA gene. Significant differences in beta diversity were found between the disease groups and the HC group in the three compartments. *Peptoniphilus, Prevotella* and *Anaerococcus* were abundant in the gut in the disease groups. Specifically, *Streptomyces, Thermus* and *Pseudomonas* showed high differential expression in both the nasal and skin compartments of children in the disease groups. These findings could provide new insights into the prevention and treatment of allergic diseases through the regulation of specific microbiota in the future.

## Data Availability

The raw data supporting the conclusions of this article will be made available by the authors, without undue reservation.
